# Supplementary material for: Are carbon emissions trading and green financial instruments synergistic? -Comprehensive quantitative research based on content analysis
Source: PLoS One. 2024 Mar 7;19(3):e0298601. doi: 10.1371/journal.pone.0298601 (PMC10919601; doi:10.1371/journal.pone.0298601)
Supplement: S1 Data — (ZIP) [file pone.0298601.s001.zip › Data/地级市Z_F工作报告.zip!/╝¬┴╓/░╫╔╜╩╨/└┤╫╘╣1⁄2╓┌║┼í╛┬φ┐╦╩2╛▌═°í┐_2009.docx]

各位代表：

　　我代表白山市人民政府，向大会作政府工作报告，请予审议。并请各位政协委员及其他列席人员提出意见。

　　一、真抓实干，攻坚克难，2008年经济社会发展实现新突破

　　年初以来，在省委、省政府和市委的正确领导下，全市上下努力践行科学发展观，突出项目建设、招商引资、民生民利等重点工作，积极应对国际金融危机带来的挑战，努力破解市场需求萎缩、资源类产品价格下滑等困难，保持了经济社会持续快速发展，圆满完成了市六届人大一次会议确定的各项任务。

　　——致力于加快发展，综合实力明显增强。经济发展呈现出速度加快、效益提高、结构优化、后劲增强的良好态势。经济总量实现了历史性突破。预计全年实现地区生产总值300亿元，同比增长20%。完成全口径财政收入30亿元、地方级财政收入15亿元，分别增长43.5%和40%。财政支出50亿元，增长20%。工业经济速度和效益同步提高。预计全年完成规模以上工业增加值120亿元，增长23%。实现利润15亿元，增长38.9%。完成技改投资85亿元，增长40%。新增规模以上企业59户，达到329户。实现新产品产值17亿元，增长45%。民营经济主营业务收入完成315亿元，增长42.5%。现代特色农业发展步伐加快。预计全年实现农林牧渔业总产值59亿元，增长9%。省级以上龙头企业发展到28户，开工建设15个千万元以上农业产业化项目，“一村一品”专业村达到46个，建设规范化特产生产基地100个。提前一年完成市区百公顷蔬菜基地建设任务。第三产业规模进一步壮大。预计全年实现第三产业增加值98亿元，增长21%。接待国内游客219万人次，旅游总收入15.6亿元，分别增长15%和25%。实现全社会消费品零售总额92亿元，增长18%。项目建设扎实推进。全年完成固定资产投资315亿元，增长48.7%。开工建设3000万元以上项目268项，其中新开工项目占全部在建项目的47%。板庙子黄金勘察开发、金刚水泥、丽美坚实木复合地板、康师傅矿泉饮品一期等105个项目竣工投产，八宝工业园、天安镁业、松江河梯级电站、东圣焦化6万吨甲醇等163个在建和新开工项目进展顺利，长白山国际旅游度假区、靖宇核电站、华能煤矸石发电等重大项目前期工作取得突破性进展。

　　——致力于改革开放，发展活力明显提升。积极探索，大胆实践，进一步激发发展活力。各项改革纵深推进。全市完成厂办大集体企业改制95户，2.5万名职工全部解除劳动关系，发放补偿金1.49亿元。国有企业改革善后工作稳步推进，部分改制企业金融债务、职工工资和社会保险等问题得到妥善解决。行政审批权相对集中改革走在全省前列，建立了重点项目审批绿色通道，缩短了审批办结时限。集体林权制度改革平稳启动。严格规范经营性土地出让制度，对全市探矿权进行清理整顿，清除140项勘察项目。招商引资成效显著。坚持走出去与请进来相结合，积极参加省组织的苏沪浙、东博会等大型经贸活动，有针对性地组织了赴珠三角、长三角、环渤海地区的招商活动，成功举办了第五届国际矿泉节、第22届长白山人参节和首届松花石文化旅游节、长白边境贸易商品交易会，引进了上海大山合、天津天士力、中国华能、东北大学产业集团等战略投资者。预计全年引进市外资金138亿元，增长73%。实际利用外资9000万美元，增长24%。完成外贸出口总额1.9亿美元，增长23%。

　　——致力于基础建设，发展环境明显优化。坚持规划先行，加大投入力度，区域环境不断提升。城市功能日趋完善。投资32亿元，开工建设了一批基础设施项目。矿欣路公铁立交桥开工建设，靖宇路、通江路公铁立交桥竣工通车，完成了24条道路工程。长白山满族风情园、地下人防商场、翠柏广场一期工程和浑江两岸江堤、主要街路亮化美化工程基本完成，市区人均新增绿地1.9平方米。新增集中供热能力80万平方米、管道燃气用户1.2万户。完成两座净水厂改扩建及7.7公里供水管网改造工程。新建商业住宅楼36.6万平方米，城市棚户区和煤矿棚户区改造完成102万平方米，安置居民10220户。完成松江河林业棚户区改造一期工程。城市污染减排工作成效显著，化学需氧量、二氧化硫削减量超额完成省定指标，全年空气优良级别天数达到307天，比去年增加27天。农村基础设施较大改善。投资6.6亿元，完成农村基础设施和社会公益事业项目361个，137个村基本实现“四清四改”，10430户农村泥草房改造顺利完工，建设农村安全饮水工程79处，解决了5.5万农村人口吃水难问题。交通建设步伐加快。全年预计完成交通建设投资13亿元。白山长白山机场实现通航，马鹿沟至八号闸边防公路竣工通车，老岭至临江、石人至新开段公路基本通车，环长白山旅游公路完成路基工程，长松岭隧道及松江河至长白公路、营城子至松江河和通化经白山至抚松高速公路共用段开工建设。完成靖宇至松江河铁路、三道沟至集安界边防公路、露水河至狩猎场旅游公路的前期工作。新修农村公路700公里，433个村实现通油路，占村总数的87%。

　　——致力于社会发展，人民生活明显改善。注重统筹协调发展，社会事业整体推进，人民群众得到了更多的实惠。各项社会事业长足发展。不断加大对社会事业的投入，中国人参博物馆、市广电大楼、体育场综合训练楼、市流浪乞讨人员救助管理站、农家书屋等一批社会公益性工程全面完工。完善和落实了农村义务教育经费保障机制，全部免除了城市义务教育阶段的学杂费。长白山职业技术学院正式成立并完成首批招生。公共卫生服务体系不断完善，新农合参合率达到80.6%，首批16个社区卫生服务中心全部建成并通过省级验收。完成了8.5万户数字电视整体转换和262个自然村广播电视“村村通”工程。“长白山采参习俗”被国务院批准为国家级非物质文化遗产。开展了喜迎奥运、绿色矿泉等系列大型文化活动，丰富了群众文化生活。成功举办了全市第六届运动会，承办了全国自行车冠军赛，在全国第十一届冬运会上获得2枚金牌、1枚银牌和3枚铜牌的优异成绩。加强了与大连理工大学、吉林大学等36所高等院校和科研单位的合作，45个科技项目获得国家、省批准立项。军民共建工作成效显著，我市被命名为全国双拥模范城。依法加强宗教事务管理，切实维护了民族团结。全面落实人口和计划生育政策，人口自然增长率控制在1.47‰。人民生活进一步改善。预计城镇居民人均可支配收入和农民人均纯收入达到13100元和4800元，分别增长13.5%和12%。扎实开展全民创业促就业系列活动，统筹城乡就业试点和小额担保贷款工作走在全省前列。全年新增就业3.9万人，下岗失业人员再就业2.5万人，农村劳动力转移1万人。社会保障体系进一步完善，全市参加养老保险人员达到20.1万人，发放离退休人员养老金10.9亿元。参加失业保险人员达到13.4万人，发放失业保险金1932万元。医疗、工伤、生育保险参保人数分别达到60万人、15万人和13.5万人。社会救助能力进一步增强。城市低保实现了动态管理下的应保尽保，为17万人发放低保资金3亿元。农村特困人口全部纳入低保范围，共为3.6万农村低保对象落实低保补助资金2158万元，人均年救助标准由360元提高到540元。助残、助学、助医、助孤等工作得到全面加强。广泛开展献爱心活动，向四川灾区捐款捐物累计2446万元，对口援建黑水县洛多乡工作扎实推进。20件民生实事基本完成。年初确定的20件民生实事有17件超额完成任务，有3件由于资金没有完全到位、调整规划设计等原因，没有全部完成，将在明年工作中予以解决。

　　——致力于民主和谐，政府自身建设明显加强。自觉接受人大的法律监督、工作监督和政协的民主监督，共办理人大代表议案和政协委员提案139件。全面落实《行政许可法》，依法对行政审批项目进行清理，市本级行政许可和非行政许可项目由416项减少到365项。加强了行政效能监察和审计监督，进一步规范了行政行为。深入开展“五五普法”教育，提高了全民法律意识。人民调解和法律援助工作不断加强。村民自治和城镇居民社区自治制度得到巩固和提高。制定并实施了市长电话值班、会议旁听、新闻发布、政务信息公开、公开征集民意“五项制度”，保障了群众的知情权、参与权和监督权，进一步畅通了政府与人民群众的沟通渠道。认真开展民主评议软环境和政行风活动，加大了涉软案件的查处力度，经济发展软环境不断改善。认真落实责任追究和领导包案制度，集中处理了一批信访积案，保持了信访形势稳定。以奥运安保为重点，加强治安防控体系建设，加大对法轮功等邪教组织的打击力度，组织开展了网上追逃、命案攻坚等专项行动，刑事发案率连续六年下降，人民群众安全感普遍增强。进一步加强煤矿、非煤矿山、危险化学品、道路交通、消防等专项安全整治，强化食品药品监管，切实保证人民生命财产安全。加强森林管护和生态建设，实现连续29年无重大森林火灾。加强应急救援演练和应急救援队伍建设，快速反应和应急处置能力不断提高。加强边境管控，保持了边境安全稳定。

　　一年来，工商、税务、金融、技术监督、统计、地震、气象、外事、民兵预备役、老龄、妇女儿童、残疾人等工作都为全市经济社会发展做出了积极的贡献。

　　各位代表，即将过去的2008年，是白山经济社会发展步伐明显加快、发展质量明显提高、发展环境明显优化、人民生活明显改善的一年。这是省委、省政府和市委正确领导的结果，是市人大、政协及各位人大代表、政协委员监督支持的结果，是社会各界鼎力支持的结果，更凝聚着全市人民的智慧和辛勤汗水。在此，我代表白山市人民政府，向全市各族人民，向驻白山的中省属企事业单位、人民解放军指战员、武警部队官兵、公安司法干警，向各民主党派、工商联、人民团体、无党派人士、离退休老同志，向关心和支持白山发展的各界朋友，表示崇高的敬意和衷心的感谢！

　　在肯定成绩的同时，我们也清醒地认识到一些不容忽视的矛盾和问题，主要是：项目超前谋划和招商力度不够，技术含量高、市场前景好、有牵动作用的大项目少。城市建设和管理还有很多薄弱环节，环境卫生、市容市貌、社区物业管理尚需进一步加强。县域经济发展不快，城乡居民收入水平不高，部分群众生活仍然比较困难。全社会亲商扶企意识不强，服务型政府建设与快发展、大发展的要求仍有一定差距。对此，我们要采取有效措施，认真加以解决。

　　二、负重奋起，戮力拼搏，推进2009年经济社会又好又快发展

　　当前，国际金融危机日益蔓延，世界经济快速衰退，明年经济将面临前所未有的困难。为有效应对危机，国家采取了更加积极的财政政策和适度宽松的货币政策，出台了进一步扩大内需、加大基础设施投入、鼓励出口创汇、刺激经济增长等若干措施。我市目前正处于加快经济转型和结构调整的关键时期，在生态建设、循环经济、基础设施、民生工程等方面将得到国家更多的支持，存在着较大的产业升级空间和巨大的发展潜力。因此，要积极应对，变压力为动力，化危机为机遇，以更积极的态度、更有力的措施、更务实的作风，全力推进白山经济社会又好又快发展。

　　经济社会发展主要预期目标是：全市地区生产总值增长18%以上，全口径财政收入、地方级财政收入增长20%以上，全社会固定资产投资增长40%以上，城镇居民人均可支配收入、农民人均纯收入分别增长12%和11.5%，城镇登记失业率、人口自然增长率分别控制在4.6%和4.1‰以内。

　　为完成上述目标，必须全力以赴抓好八项工作。

　　（一）全力推进工业强市，更加注重结构和效益，在扩充经济总量上实现新提升

　　面对市场需求低迷、资金短缺加剧的严峻形势，我们必须付出更加艰苦的努力，坚持一手抓外延扩大增量、一手抓内涵提升存量，保持工业经济平稳较快增长。全市规模以上工业总产值增长25%，增加值增长23%，实现利润10亿元以上。

　　加快项目建设扩大增量。坚持把项目建设作为经济工作的重中之重，紧紧抓住国家加大投资、扩大内需的有利机遇，突出投资拉动，全力以赴抢上一批左右全市经济形势的大项目，推进产业结构优化升级，支撑经济快速发展。全年完成固定资产投资440亿元，工业固定资产投资要占全社会投资总额的50%以上。计划安排3000万元以上重点项目330项，完成投资310亿元。加快推进180个续建项目，八宝工业园、琦祥纸业二期工程、东锋镁业等86个项目竣工投产；千方百计落实开工条件，华能煤矸石发电、生物质发电、大山合食用菌综合开发等105个新项目开工建设；积极抓好重大项目的前期准备，靖宇核电站开始移民和“五通一平”临建施工，加快推进鸭绿江界河电站等项目的前期工作。

　　加快扩能改造提升存量。深入开展“工业企业成长年”活动，推动骨干企业做大做强。建立存量骨干企业运行动态数据库，对销售收入超亿元的喜丰集团、东圣焦化、天风制药等70户骨干工业企业进行重点跟踪服务，逐户研究解决生产经营中的困难和问题，不断加大资金、煤电油运等要素的协调配置力度，促进存量企业加快技术改造、产品开发和扩大规模，最大限度释放产能。启动“百户企业成长工程”，重点扶持和培育100户成长性好的企业，力争八道江健宁药业、抚松仁义铁矿等50户企业达到规模以上标准。切实加强企业能力建设，继续实施技能型人才培养、万名小老板创业和千名企业家培训工程，引导企业加强管理，建立现代企业制度，增强企业的管理、营销、融资和创新能力，降低经营管理成本，提高市场竞争力。全年完成技术改造投资102亿元，增长20%，实现新产品产值22亿元，增长30%。申报省级以上科技发展项目20项，力争培育10户科技孵化企业。坚持放手、放开、放活，大力发展民营经济，实现主营业务收入增长20%以上。

　　加快特色园区建设。立足特色资源优势和产业基础，推进经济开发区、工业集中区和特色园区建设。明确功能定位，完善规划，加大水、电、路、标准厂房等基础设施投入，不断增强园区的承载能力。鼓励引导产业关联度高、成长性好的企业入驻园区，推动产业集聚和要素集中。以琦祥纸业为依托，开工建设包装工业园，逐步形成以造纸、纸制品、商标彩印、物流配送于一体的多功能产业园区。启动光伏产业园和长白山生态健康科技产业园区建设，力争引进锦州阳光能源集团、天津天士力集团等有实力的大企业入园进区，提升产业层次。

　 （二）全力推进改革开放，更加注重广度和深度，在增强发展活力上谋求新突破

　　继续深化改革，进一步扩大对外开放，增强经济发展的动力与活力。

　　稳步推进各项改革。基本完成厂办大集体改革试点工作。深化农村改革，加快土地有偿流转，促进适度规模经营。加快粮食购销企业产权制度改革。全面启动集体林权制度改革，大胆探索地方国有林场改革和森林资源有偿流转。推进政府机构改革和行政审批权相对集中改革。按照国家统一部署，认真做好增值税转型、燃油税改革工作。

　　强力推进招商引资。紧紧抓住国内外资本避险和产业转移的有利时机，在全市广泛开展“招商引资年”活动，全民齐动员，举市大招商，全年力争引进市外资金180亿元以上。一是实施产业推进组招商。积极发挥八个产业推进组的作用，谋划生成一批带动能力强、产业关联度高、成长性好的大项目。加强与世界500强企业及国内百强企业的高位对接，有针对性地开展重大项目招商。二是实施重点区域小分队招商。抽调骨干力量成立高层次、专业化的招商小分队，以新加坡、韩国、香港及京津鲁、长三角、珠三角等地区为招商重点，实行叩门招商，提高招商成功率。三是实施委托代理招商和挂职招商。充分挖掘和广泛密切与友好城市、区域经济协作组织的联系，搭建合作桥梁，在全国发达城市和有合作关系的国外城市，设立招商网点，聘请招商顾问，实行委托代理招商。选派懂经济、有潜力的优秀后备干部赴经济发达地区经济部门和有关商会挂职招商，确保招商实效。四是实施节庆活动招商。积极参加省组织的东博会等经贸活动，办好旅游节、矿泉节、人参节等节庆活动，促成更多的项目和资本落户白山，提高招商项目的签约率、履约率和资金到位率。五是实施全民招商。发挥广大干部群众的主观能动性，充分利用亲缘、血缘、地缘关系，以亲联商、以情感商、以商招商，掀起全民招商热潮。

　　加快推进对外开放。不断扩大对外交流与经贸合作，提高经济外向度。全年完成外贸进出口总额2.4亿美元，增长14.3%。抓住国家提高出口退税率的时机，积极协调解决企业出口受阻、融资保险、通关检疫等方面的问题，鼓励引导企业把外贸代理业务转为自营进出口，培育新的外贸增长点。加大政策扶持力度，扶持金隆木业、丽美坚等企业扩大出口，引导人参、硅藻土等出口生产企业加大新产品研发力度，鼓励企业积极申报出口名牌，增强国际市场竞争力。组织企业“走出去”开拓国际市场，积极推进对俄、朝木材深加工和矿山开发项目。加强与俄罗斯、韩国、日本等国家各类劳务组织的沟通和联系，促进劳务输出和境外工程承包。加快长白边境贸易区和临江口岸建设，促进对朝边境贸易平稳增长。

　 （三）全力推进现代特色农业发展，更加注重农业增效和农民增收，在新农村建设上再上新台阶

　　坚持用工业化思维谋划农业和农村经济发展，促进农业增效、农民增收、农村环境改善。

　　突出发展现代特色农业。坚持标准化生产、规模化经营，重点抓好参、蛙、菌、药等特色农产品基地建设，新建“一村一品”专业乡镇3个、专业村9个。高标准制定人参产业发展规划和市场准入标准，建设人参精深加工产业园，逐步缩减园参留存面积，扩大林下参种植规模，认真抓好40个人参标准化小区建设，推广人参GAP标准化栽培面积1500万平方米，提升人参产业精深加工能力，参业销售收入力争增长20%以上。大力发展以长白山特种野猪为重点的特色精品畜牧业，特种野猪饲养量达到5万头，建设全国最大的特种野猪养殖加工基地。建成国家级农产品质量检验中心，新增有机、绿色、无公害农产品认证50个。全年实现农业总产值63亿元，其中特产业产值达到43亿元，增长20%。加快农业产业化进程，扶持一批龙头企业做大做强，省级以上龙头企业发展到30户，市级龙头企业达到60户。新增20个村级农产品加工企业和20个村产业化规模基地。市区新建蔬菜温室100栋、大棚100栋，发展露地蔬菜1000亩。

　　扎实推进新农村建设。深入实施县域突破战略，坚持发展政策向县域倾斜，发展要素向县域集中，突出特色，发挥优势，不断壮大县域经济整体实力。实施“百村万户”升级工程，在全市选择100个村、1万个农户，实行生产项目小额贷款扶持，年贷款额不少于3亿元，人均增收1000元。全面抓好农民培训工作，农民技能培训达到3.5万人次，转移农村富余劳动力5万人。规范400个新型农村经济合作组织，发挥各类农村专业协会和农村经纪人作用，提高农业市场化组织程度。改造农村泥草房9940户，基本消灭农村危房户。新建沼气池1000个。加快村村通油路工程建设，新修农村公路500公里，通油路行政村达到438个。加强公路、铁路、边境和旅游沿线村屯卫生环境整治，150个村完成“四清四改”。继续抓好广播电视“村村通”工程，加快农村电网、通讯网络、信息站等建设。

　　（四）全力推进基础设施建设，更加注重构筑特色和完善功能，在增强城市承载能力上有新提高

　　按照“构筑特色，营造绿色，打造精品，提升魅力”的理念，扎实推进“四城联创”工作，努力建设功能完善、管理高效、环境优美、和谐文明的新白山。

　　高起点高层次规划城市。进一步完善城市总体规划，增强规划的前瞻性和科学性，搞好与国家主体功能区、产业发展、土地利用、重大基础设施等规划的衔接，完成长白山大街、启明路、浑江两岸等重点区域控制性详规。

　　高质量高品位建设城市。实施“六大工程”。一是道路畅通工程。矿欣路公铁立交桥、向江大桥主体完工，改造加固彩虹桥，新建南平街、自强街、矿源路、喜丰路等30条街路，开工建设向阳路、南康路、靖宇路三条南北贯通路，改造通往长春、通化、临江方向三个城区出口，做好北外环道路前期工作。二是绿化工程。抓好站前、文化等7个广场及北山、江源等3个公园绿化工作，绿化改造铁路沿线、浑江沿岸，新增绿地面积50万平方米，新植树木10万株。三是美化工程。新建江北、向江、南岭、通沟集会休闲广场，续建翠柏广场，在满族风情园、江源公园等处建造7座城市雕塑。四是亮化工程。对浑江大街、长白山大街等主要街路和浑江两岸、希望广场、政府广场及广电大厦、地旺商厦等标志性建筑实施亮化。五是旧城改造工程。建设长白山特产城、隆府大厦、合兴商贸物流中心等高层建筑，并对周边旧区进行改造。六是净化工程。完成污水处理厂主干网铺设和江北新区1500米配套管网建设，开工建设江源污水处理厂，在市区、旅游景区新建高标准公厕28座，维修公厕30座。

　　高效能高水平管理城市。全面开展“城市管理年”活动，大力开展文明白山、和谐白山主题教育，抓好文明单位、文明社区、文明校园、文明家庭等创建活动，从源头上遏制不文明行为。加强城市环境综合整治，通过开展交通秩序、环境卫生等专项整治活动，着重解决红旗贸易城、山货市场等城市中心区域环境脏、乱、差和城市噪声、烟尘污染问题。下大力气取缔马路市场、店外店和流动摊点，规范广告牌匾，切实解决私搭乱建、乱停乱放、乱贴乱画和损坏公共设施等问题。强化小区物业管理，提高社区服务水平。

　　高站位高速度加快交通建设。按照“抢上高速，快上铁路，打通边境，完善基础”的工作思路，加快经济主干路、旅游路、边境路、城乡便民路建设，全年完成投资10亿元以上。力争营城子至松江河、通化经白山至抚松高速公路全线开工建设，环长白山旅游公路、老岭至临江、石人至新开段公路和市区连接线工程竣工通车。开工建设靖宇至松江河铁路、三道沟至集安界边防公路、露水河至狩猎场旅游公路。加快推进白山至长春、白山至临江、松江河至长白高速公路、市区外环路及苇沙河至错草边防公路项目的前期工作，逐步完善集航空、高速、铁路于一体的立体交通网络体系。

　　（五）全力推进旅游产业加快发展，更加注重提高层次和服务水平，在促进现代服务业发展上迈出新步伐

　　以创建中国优秀旅游城市为目标，突出发展旅游业，加快发展服务业，努力构建以旅游为牵导的现代服务业体系。

　　大力发展旅游业。完成全市旅游总体规划修编，做好重点景区景点详规，加快旅游资源整合，打造松花江上游风光带、鸭绿江风光带、环城景观带，构建“环山、沿江、跨国”的旅游发展格局。加快旅游项目建设，聘请专业策划公司精心包装一批高端旅游项目。全力推进长白山国际旅游度假区建设，力争早日把白山打造成中国旅游名市。举办长白山鸭绿江边境风情旅游节，突出生态观光、休闲度假、边境风情、民族风俗、红色旅游，精心设计旅游精品线路，加快龙山湖、望天鹅、露水河国际狩猎场等一批景区、景点基础设施建设，积极组织自驾车游等赛事活动，倾力打造区域性旅游节庆盛会。提升旅游配套服务水平，长白山温泉度假休闲中心、雨润长白山国际大酒店、温泉度假产权公寓年内竣工投入使用，加快推进长白山香洲大酒店、南林大酒店等一批四星级以上宾馆建设。加强旅游市场和安全秩序的监督检查，规范定点一批旅游宾馆酒店，制定接待标准，抓好服务培训和监管，提高旅游从业人员服务水平。积极开发松花石、长白玉、黑陶等具有长白山特色的旅游纪念品。大力推进旅游营销创新，突出特色、抓住看点、宣传亮点，通过新闻发布会、央视宣传片、广告牌及画册等多种形式，大力宣传推介白山旅游资源。全面加强与国内知名旅游企业的联系合作，积极参加旅游交易会、展销会，不断扩大白山在国内外旅游市场的影响力。

　　积极发展现代服务业。完善市场流通体系，促进消费需求快速增长。全年实现社会消费品零售总额109亿元，增长18%。优化商业网点布局，推进城乡各类批发市场、特产专业市场建设，苏州商贸城、地下人防商城投入使用。鼓励商业企业创新运营模式，积极引进国内外知名连锁经营企业。加快发展第三方物流，建立和完善综合性与专业性并存的多层次物流配送体系，打造具有地域特色的商贸中心和综合物流园区。加快无线移动通讯网络建设，提高无线通讯网络覆盖率。积极发展资产评估、咨询服务等中介机构和金融、保险、信息、会展等新兴服务业，推进现代服务业加快发展。

　　（六）全力推进各项社会事业发展，更加注重民生和保障，在和谐社会建设上再创新业绩

　　加快推进以改善民生为重点的社会事业建设，扩大公共服务，完善社会管理，促进社会公平，使广大群众在改革发展中得到更多实惠。

　　大力发展各项社会事业。继续加大教育投入，全面落实免除农村和城市义务教育学杂费政策，推进教育均衡发展。加快长白山职业技术学院、骨干职业学校建设。深化医疗卫生体制改革，加强公共卫生、医疗服务体系建设，完成市120急救中心建设，城市社区卫生服务中心全部达标，市中心区社区卫生服务覆盖率达到100%。新型农村合作医疗参合率达到82%，年人均筹资标准提高到100元。继续稳定低生育水平，提高出生人口素质。深入贯彻党的民族宗教政策，促进少数民族和民族地区经济社会发展。推动具有长白山地域特色和满族民族特色的文化产业发展，长白山满族文化博物馆向社会全面开放。承办好第五届全省社区体育健身大会，全力备战第十六届省运会。全面完成数字电视整体转换任务。

　　完善就业社会保障体系。全面落实就业政策，推进城乡统筹就业试点工作，实行订单式就业培训，积极筹建技师学院，扎实开展全民创业促就业活动。全年新增城镇就业3.4万人，免费培训下岗失业人员1万人。进一步完善社会保障制度，加大养老、失业、医疗、工伤和生育保险扩面征缴，重点推进非公经济组织人员参加养老保险和矿山、建筑企业参加工伤保险。加快推进新征地农民养老保险工作。全年新增养老保险1万人、城镇居民医疗保险2万人、工伤保险1万人。强化劳动监察，畅通维权投诉机制，实行部门联动，重点解决拖欠农民工工资、不执行最低工资标准和不依法参保等问题，切实维护劳动者的合法权益。对特困群体、重病重残人群、流浪乞讨人员、孤寡老人等弱势群体提供社会救助，市儿童福利院和残疾人康复就业服务中心投入使用。

　　抓好社会稳定工作。继续开展“平安白山”创建活动，加强社会治安综合治理，深入开展“打黑除恶”等专项行动，依法打击各类违法犯罪，力争刑事案件破案率居全省前列，各类可防性案件稳中有降，命案侦破率在90%以上。严厉打击邪教组织，坚决遏制非法宗教活动。进一步完善党政军警民合力治边的有效机制，维护边境稳定。完善矛盾纠纷排查化解长效机制，集中精力解决涉及民生的信访问题，妥善处置社会矛盾和群体性事件，最大限度减少不和谐因素。加强煤矿、非煤矿山、食品药品、道路交通、公众聚集场所消防等公共安全专项整治，坚决遏制重特大事故发生，确保人民群众生命财产安全。

　　（七）全力推进生态文明建设，更加注重环境保护和节能减排，在推进可持续发展上取得新成效

　　坚持生态立市，正确处理发展与保护的关系，加大对环境保护的投入力度，大力发展循环经济，严格节能减排，切实提高可持续发展能力。

　　加大生态环境保护力度。加强森林资源经营和管护，提高森林质量和林地产出水平，严厉打击各类涉林违法犯罪。突出抓好森林防火，确保实现连续30年无重大森林火灾。实施生态造林，抓好城区环山绿化，完成植树造林2.5万亩、封山育林10.5万亩。加强松花江上游、鸭绿江、浑江干流水污染治理。加快矿山开采区的综合治理，逐步恢复生态功能。积极争取国家生态环境补偿专项资金和衰退产业援助政策，有计划地实施生态移民。

　　大力发展循环经济。按照“减量化、再利用、资源化”的原则，加强资源能源节约和综合高效利用。对矿泉水、煤炭、铁矿石、硅藻土等重要资源，实行统筹规划，有序集约开发，搞好精深加工，提高产品附加值。综合利用煤矸石、粉煤灰、铁矿石尾矿、木材“三剩物”等废弃资源，变废为宝。坚决关闭工艺落后、耗能高、污染大的企业，重点实施一批节能技术改造项目，对金刚水泥余热发电、东锋镁业还原炉改造及余热利用、东圣焦化6万吨甲醇项目加强跟踪管理，确保如期完工。对综合能源消费量5000吨标准煤以上的企业，下达节能考核指标，未达标的收取超能耗保证金，强制进行节能改造。积极开展国家环保模范城市创建工作，深入开展节能降耗全民行动，增强全社会节能环保意识。全市万元工业增加值综合能耗降低3%，削减化学需氧量、二氧化硫2%以上。

　　（八）全力推进财税金融工作，更加注重增收节支和金融服务，在壮大地方实力上取得新成果

　　继续抓好财源培植和财税征管，狠抓增收节支，积极引导金融信贷资金服务区域经济发展。

　　开源节流促增收。继续推进部门预算、国库集中支付、非税收入管理体制等财政制度改革，建立符合市场经济体制的公共财政体系。充分发挥财税的杠杆作用，推动项目建设，培植新增税源，增强财税后劲。强化税收征管措施，大力开展综合治税，全面普查税源，准确掌握税源分布状况，加强重点行业、重点企业、重点区域税源管理，保证主体税源持续增长。严厉打击各种偷逃骗漏税行为，堵塞税收流失漏洞，整顿和规范各种行政事业性收费、政府性基金和罚没收入，做到应收尽收。进一步推进国有资产经营，完善国有资产监管体制，建立国有资产营运平台。用足用好国家财政扶持和债务减免政策，在向上争取政策和资金方面有更大突破。硬化预算约束，大力压缩行政事业经费和一般性支出，提高资金使用效益。

　　强化金融服务保障。不断优化金融生态环境，鼓励支持金融机构提供更多灵活的融资业务和金融产品。拓宽中小金融机构融资渠道，争取2家以上法人金融机构加入全国银行间同业拆借市场。开办中小企业短期融资业务，对符合条件的企业发放短期融资债券。增加全市中小企业担保公司资本金注入，壮大担保能力。积极探索应收账款、林权、土地等抵押贷款方式，拓宽担保领域。争取再组建2家担保公司和3家以上小额贷款公司。积极扶持企业横向联合，寻求域外企业参股融资。积极发挥国投公司化解金融债务的作用，打包回购714户企业49亿元金融债权。进一步推进企业上市，启动金隆木业、东圣焦化上市股改程序和上市辅导，做好东锋镁业上市申报。

　　三、务实创新，奋发有为，努力建设人民满意政府

　　要切实转变政府职能，改进工作作风，推进管理创新，不断提高执行力和公信力，全力打造服务型、诚信型、学习型、廉洁型政府。

　　（一）坚持以人为本，努力建设为民利民政府。要满怀爱民之心，常思为民之责，多办利民之事。明年新增财力的70%以上用于公共事业和民生方面支出，努力办好十件实事：一是投资37.4亿元，完成城市棚户区140万平方米、煤矿棚户区90万平方米、沉陷区异地建设19.8万平方米住宅楼建设，安置居民2.65万户。全面启动林业棚户区改造工程。二是投资1.4亿元，新建廉租住房10万平方米，解决2000户无房户。三是投资2000万元，启动西北岔第二水源地工程，年内完成初步设计和“三通一平”。四是投资1000万元，启动市区67座供水泵站、3万户供水支线管网改造工程。投资1400万元，完成36处农村安全饮水工程，解决3.5万人饮水困难。五是投资3000万元，市区新增供热能力60万平方米，新增燃气用户1.2万户。六是启动碱厂沟河、月牙河低洼内涝综合治理工程。完成浑江干流青沟子河口至木锨沟河段浆砌石护岸工程。七是投资5000万元，移地新建市中医院并完成一期工程。八是投资4500万元，完成浑江大桥主体工程。九是投资4500万元，开工建设松江河火车站改造工程。十是城镇低保标准由月人均160元提高到180元，农村低保标准由年人均540元提高到600元。退休人员养老金月人均标准由822元提高到900元以上。

　　（二）坚持求真务实，努力建设服务高效政府。各级政府要突出服务经济、服务民生，规范服务行为，提高服务质量，切实加强社会管理和公共服务职能，为全社会提供更多更好的公共服务。进一步清理行政审批项目，实行大厅统一受理、即时办理、限时办结。继续坚持完善阳光政务“五项制度”，实施政务公开，主动接受社会监督。加强各级政府机关执行力建设，完善机关目标管理绩效考评机制，健全和落实岗位责任制，狠抓各项工作的落实。围绕全市中心工作量化目标任务，实行限时办结、督查考核等制度，对重点工作已经落实的问效果，正在落实的看进度，尚未落实的查原因，落实不利的追责任，切实提高行政执行能力。

　　（三）坚持依法行政，努力建设法治诚信政府。坚持把依法行政贯穿于政府决策、执行、监督的全过程，严格按照法定权限和程序行使权力、履行职责。自觉接受人大的法律监督和工作监督，主动接受政协的民主监督，进一步提高人大代表议案、建议和政协委员提案办理质量，认真听取各民主党派、工商联、无党派人士和人民团体的意见，自觉接受新闻舆论和公众监督。坚持科学民主决策，完善重大决策、重要事项的公开咨询、公开听证和公示等制度，实行阳光行政。加强行政复议工作，健全行政执法责任制，强化行政执法监督，大力推进严格执法、公正执法、文明执法。着力建设“诚信政府”，诚实守信，诚信办事，一经承诺坚决兑现，树立白山重发展、讲诚信的良好形象。

　　（四）坚持从严治政，努力建设清正廉洁政府。坚持标本兼治、综合治理、惩防并举、注重预防的方针，建立健全教育、制度、监督并重的惩治和预防腐败体系，切实规范行政权力运行，在财政资金使用、国有资产运营、工程建设招投标、土地使用权出让、行政审批等重点领域，建立严格的监督制度和权力制约机制，严肃查办大要案，坚决惩处腐败分子。强化行政监察和审计监督，深入开展民主评议政行风和软环境“双评”活动，坚决纠正部门和行业不正之风。

　　各位代表，新的形势催人奋进，人民重托千钧在肩，发展使命任重道远。让我们在省委、省政府和市委的正确领导下，开拓创新，奋力拼搏，为推进白山经济社会又好又快发展而努力奋斗！
